# An immune infiltration-related long non-coding RNAs signature predicts prognosis for hepatocellular carcinoma

**DOI:** 10.3389/fgene.2022.1029576

**Published:** 2022-12-08

**Authors:** Gen Li, Shaodian Xu, Shuai Yang, Cong Wu, Liangliang Zhang, Hongbing Wang

**Affiliations:** ^1^ Beijing Hospital of Traditional Chinese Medicine, Capital Medical University, Beijing, China; ^2^ Xuanwu Hospital, Capital Medical University, Beijing, China

**Keywords:** hepatocellular carcinoma, lncRNAs, immune infiltration, prognostic signature, microenvironment

## Abstract

**Background:** With a high incidence and dismal survival rate, hepatocellular carcinoma (HCC) tops the list of the world’s most frequent malignant tumors. Immunotherapy is a new approach to cancer treatment, and its effect on prolonging overall survival (OS) varies from patient to patient. For a more effective prognosis and treatment of HCC, we are committed to identifying immune infiltration-related long non-coding RNAs (IIRLs) with prognostic value in hepatocellular carcinoma.

**Methods:** In our study, we calculated immune scores of 369 hepatocellular carcinoma samples from the Cancer Genome Atlas (TCGA) database by using an estimation algorithm, and obtained long non-coding RNAs (lncRNAs) associated with immune infiltration by using Weighted Gene Co-expression Network analysis (WGCNA). For training cohort, univariate Cox, least absolute shrinkage and selection operator (Lasso) and multivariate Cox regression analysis were used to determine prognostic IIRLs, we established a prognostic IIRLs signature. By testing cohort and entire cohort, we confirmed that the signature is practical. The prognosis of people with different clinicopathological stages and risk scores were predicted by the nomogram we constructed. In addition, Immune cell infiltration analysis and prediction of therapeutic drugs were performed.

**Results:** 93 IIRLs were obtained by WGCNA. Furthermore, the prognostic value of these IIRLs were evaluated by using univariate Cox, Lasso and multivariate Cox analysis. Four IIRLs were used to create a signature with a prognosis. Time-related receiver operating characteristic (ROC) curve revealed that this model had an acceptable prognostic value for HCC patients. By using univariate and multivariate Cox regression analysis, this risk score has been shown to be an independent prognostic factor for HCC. The nomogram we made showed good predictions. Except for that, the treatment with immune checkpoint inhibitors (ICI) was likely to be more effective for low-risk patients.

**Conclusion:** Based on four IIRLs, a prognostic signature was created in this research showed good accuracy in predicting OS. This study also provided valuable references for Immunotherapy of hepatocellular carcinoma.

## Introduction

As the seventh most common cancer worldwide, fatality rate of primary liver cancer is second among cancers (Bray et al., 2018; Chidambaranathan-Reghupaty et al., 2021). Hepatocellular carcinoma (HCC) is the most frequent kind of liver cancer, accounting for around 90% of primary liver cancer, and its incidence is on the rise worldwide (Kulik and El-Serag, 2019; Llovet et al., 2021a). However, the 5-years survival rate for HCC is very low (Jemal et al., 2017). In the treatment of hepatocellular carcinoma, HCC patients are staged according to the Barcelona Clinic Liver Cancer (BCLC) staging system (Llovet et al., 1999; European Association for the Study of the Liver, 2018; Llovet et al., 2021b). Resection, transplantation and local ablation are choices for patients with incipient hepatocellular carcinoma, while transcatheter arterial chemoembolization (TACE) is the first choice for patients at intermediate stage, and systemic therapy is preferred for people with advanced hepatocellular carcinoma (Llovet et al., 2021a). The extracellular matrix, blood vessels, immune cells, neurons, and other biological activities surrounding a tumor are known as the tumor microenvironment (TME), and they have strong ties to tumor development and response to therapy (Fridman et al., 2012; Kim et al., 2020). Immunotherapy has evolved over the past decade to become a new way to treat advanced tumors (Galon and Bruni, 2019; Cherkassky et al., 2022), and Immune checkpoint inhibitor (ICI) is a type of immunotherapy. As a second-line agent for systemic therapies, ICI is applied to HCC patients. If sorafenib failed, advanced HCC may be treated with either of three authorized regimens: regorafenib, cabozantinib, or ramucirumab. In addition, nivolumab (anti-PD1 inhibitors) and pembrolizumab (anti-PD1 inhibitor) are approved by the Food and Drug Administration (FDA) as single agents, and the combination including ipilimumab (CTLA4 monoclonal antibody) and nivolumab has been given the green light (Yau et al., 2020; Llovet et al., 2021a). With an objective response to ICI regimens, the average overall survival time for HCC patients is more than 30 months (Yau et al., 2020; Llovet et al., 2022). The result of the response to ICI monotherapy is used to enhance OS endpoints in clinical trials. There has been a long-term search for predictable clinical and tissue biomarkers, but some cannot be applied clinically due to their low sensitivity (Pinero et al., 2020; Llovet et al., 2022). It is urgent for us to find immune-related biomarkers that can forecast prognosis and therapy sensitivity in HCC.

The term “long non-coding RNA” (lncRNA) refers to non-coding RNAs that are longer than 200 nucleotides but playing no major role in protein coding (Wang et al., 2011). More and more evidences have shown that lncRNAs are essential components of the immune system and have the ability to control immune cell differentiation and function in cancer (Denaro et al., 2019; Wu et al., 2020). A study about HCC shows that the lnc-epidermal growth factor receptor (lnc-EGFR) encourages the development of Treg cells and prevents the activity of cytotoxic T lymphocytes (CTLs) (Jiang et al., 2017). Another hepatocellular carcinoma study shows that lncRNA nuclear enriched transcript 1 (NEAT1) inhibits the antitumor activity of CD8+T-cell and promotes their apoptosis through the Mir-155/TIM-3 pathway (Yan et al., 2019). Such being the case, Discovering lncRNAs associated to immune infiltration that is crucial for clinical prognosis and therapy.

In our study, we used the lncRNA dataset of HCC patients in the Cancer Genome Atlas (TCGA) to create a model of prognostic risk associated with immune infiltration. We elucidated its ability to predict overall survival of HCC patients. We also analyzed the correlation between the risk score constructed on the basis of immune infiltration-related lncRNAs (IIRLs) and immune cells and predicted the potential treatment drugs with different drug sensitivity between two groups.

## Materials and methods

### Data access to hepatocellular carcinoma

The RNA-sequencing (RNA-seq) transcriptomic data and clinicopathological characteristics of liver hepatocellular carcinoma (LIHC) were downloaded from The Cancer Genome Atlas (TCGA) database (https://portal.gdc.cancer.gov/). There were 374 tumor samples and 50 nearby tissues in the original data set. The sample data and clinical information were combined, excluding the adjacent tissue samples. The analytic data set for our research, consisting of 369 samples of hepatocellular carcinoma, was finally identified. According to the data, it was divided into mRNA data and lncRNA data.

### Screening for immune infiltration-related lncRNAs

To determine the ImmuneScore for each HCC sample, we utilized the ESTIMATE package (Yoshihara et al., 2013) from the R suite. Through the lncRNA data, the WGCNA package (Langfelder and Horvath, 2008) in the R suite was used to create a scale-free co-expression network. The modules associated with the ImmuneScore were selected and the lncRNAs in the modules were extracted for subsequent analysis.

### Construction of an immune infiltration-related lncRNAs prognostic signature

369 samples were used with 185 for training and 184 for testing. According to univariate Cox analysis, prognostic relevance of IIRLs was assessed in the training cohort. LncRNAs with *p* < 0.05 were considered to be promising candidates. Using the candidate lncRNAs and the glmnet package (Friedman et al., 2010), we constructed a LASSO regression model. Making use of the survival package, we obtained the hazard ratios (HR) and regression coefficients by introducing lncRNAs into a multivariate Cox model. We applied regression coefficients to construct a risk score. The following procedure was used to calculate the risk score

$$Risk\;score=\sum_{i=1}^{n}Coefi*Expi$$

The expression of IIRLs in the signature was represented by Expi, with Coefi representing the regression coefficients. According to the median risk score, the patients were sorted into high-risk and low-risk groups. We examined the performance of prognostic indicators in terms of overall survival (OS) by using Kaplan Meier (KM) survival and receiver operating characteristic (ROC) curve analysis. Validation of the prognostic signature developed by training cohort was performed on testing cohort and entire cohort.

### Evaluation of prognostic factors and development of a predictive nomogram

KM survival analysis was carried out in two groups to clarify differences among subgroups based on gender, age, grade, and American Joint Committee on Cancer (AJCC) stages. Clinical features and risk score were applied to univariate and multivariate Cox regression analyses in order to determine whether they were independent prognostic factors. The line chart was applied to predict 1-year, 3-years, and 5-years survival rates. We used concordance index (C-index) and calibration curves to assess the stability of this model.

### Functional enrichment analysis

According to the limma package (Ritchie et al., 2015), different analysis was conducted on mRNA data to clarify the biological function differences between two groups. We used the gene set enrichment analysis (GSEA) function in clusterProfiler (Yu et al., 2012; Wu et al., 2021a) to perform Gene Ontology (GO) and Kyoto Encyclopedia of Genes and Genomes (KEGG) enrichment analysis for difference analysis results.

### Immune correlation analysis

The single-sample gene set enrichment analysis (ssGSEA) algorithm was used to assess immune-related function of each sample (Hanzelmann et al., 2013). We obtained the infiltration results of 22 immune cell subtypes in each sample by using CIBERSORT algorithm (Newman et al., 2015) from the TIMER (Li et al., 2016; Li et al., 2017; Li et al., 2020) database. We examined the variations in immune cells in two groups as well as the relationships among various immune cells. We obtained the immune cell infiltration results of different algorithms from the TIMER (29–31) database, and found out the relationship between risk scores and different immune cells.

### Evaluation of the Model’s importance in predicting drug susceptibility

The Wilcoxon signed-rank test was used to compare immune checkpoint gene expression levels between two groups for PD-1, PDL-1, CTLA-4, LAG-3, and VSIR. From the Cancer Immunome Atlas (TCIA) database (https://tcia.at/), we retrieved the Immunophenoscores (IPS) of HCC patients. We compared the IPS between two groups in various immunotherapy choices in order to predict the sensitivity of immunotherapy. Using the pRRophetic package, we tallied the half-maximal inhibitory concentration (IC50) of standard chemotherapy and molecular targeted medicines for each sample to assess the signature’s utility in predicting the success of HCC treatment (Geeleher et al., 2014a; Geeleher et al., 2014b). Wilcoxon signed-rank test (*p* < 0.001 as significant level) was used to compare the IC50 between two groups. We used the spearman rank correlation to determine whether there was a connection between risk scores and IC50 (with a cutoff of *p* < 0.001 and an absolute value of the Spearman rank correlation coefficient |R|≥0.25).

## Results

### Identify immune infiltration-related lncRNAs

**FIGURE 1:**
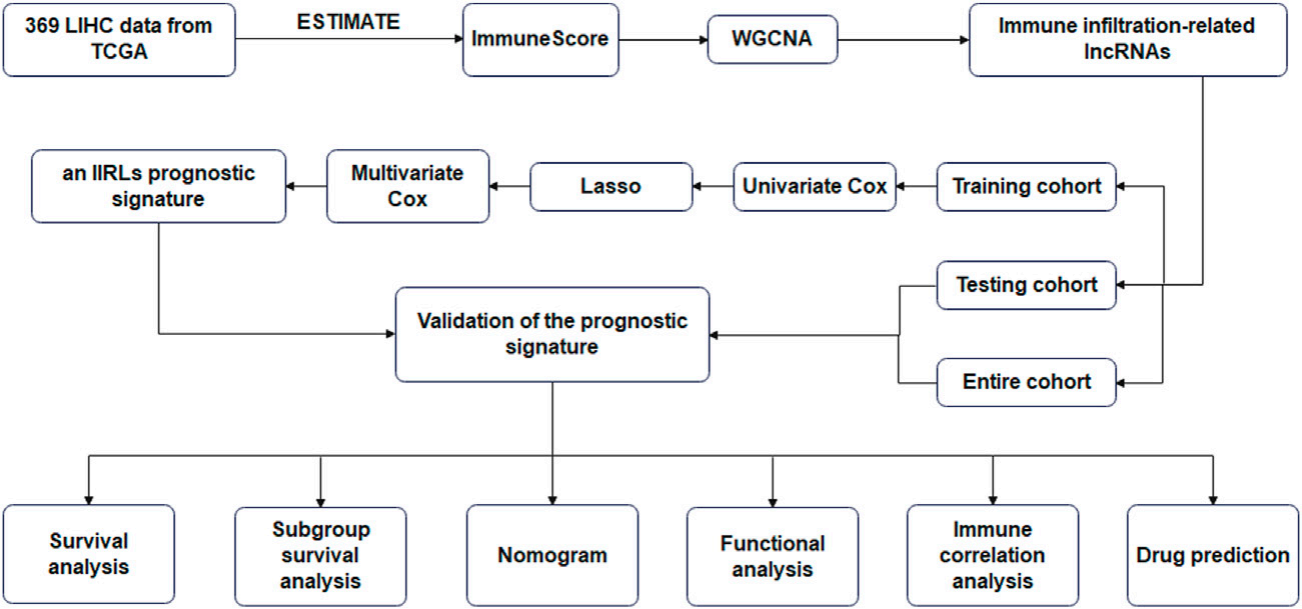
The flow chart of this study.

**FIGURE 2:**
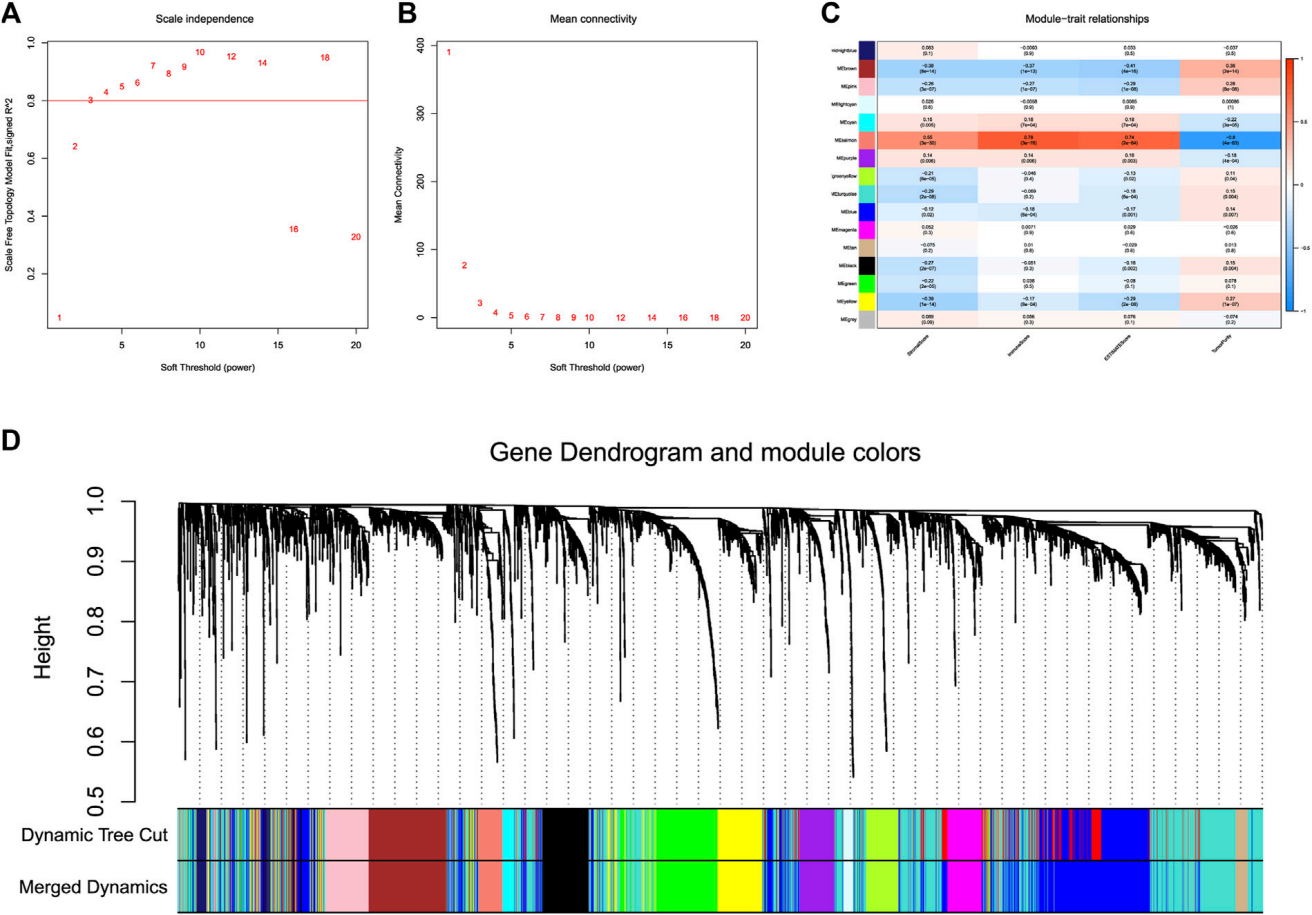
WGCNA of lncRNAs in HCC. **(A)** The scale-free fit index for different softthresholding powers is analyzed. **(B)** Analysis of the mean connectivity for different soft-thresholding powers. **(C)** ImmuneScore and its inter-module correlations shown as a heatmap. **(D)** Clustering dendrogram of 369 hepatocellular carcinoma samples.

The flow chart of study was shown in Figure 1. 369 LICH sample data were filtered out of low-expression RNA-seq data and converted into mRNA data and lncRNA data according to the data. We used WGCNA to analyze lncRNA data. We selected a soft threshold of 3 (Figures 2A,B) and chose 30 as the minimum number of lncRNAs in the module. We set the threshold to 0.25 of cutting height to combine any potentially similar modules, resulting in 16 modules (Figure 2D). The salmon module was highly positively correlated with ImmuneScore (correlation coefficient R = 0.78, *p* < 0.001) (Figure 2C). Thus, we obtained 93 IIRLs.

### Construction of an immune infiltration-related lncRNAs prognostic signature

**FIGURE 3:**
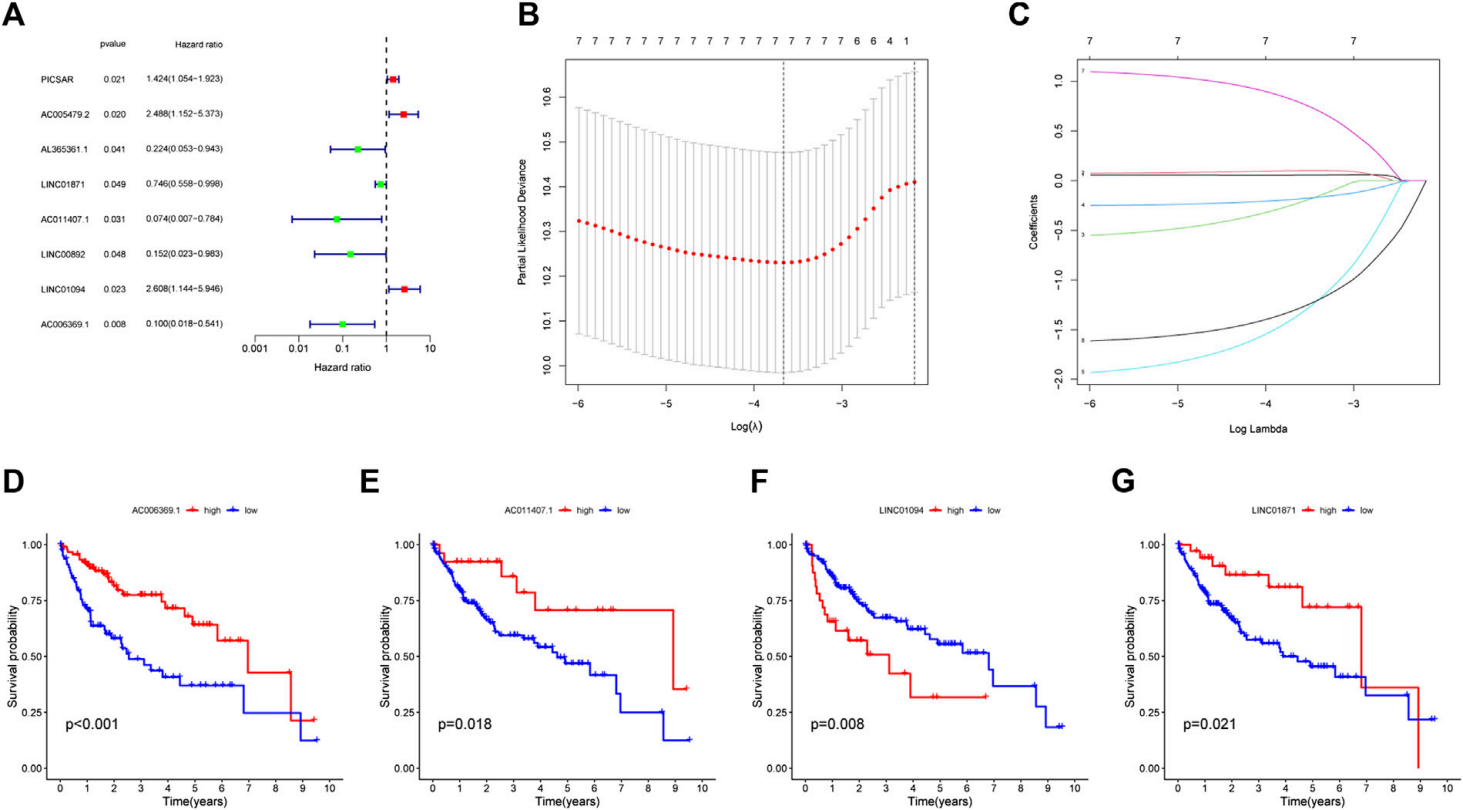
Selection of IIRLs and KM survival curves for the four prognostic IIRLs. **(A)** Univariate Cox regression showing eight IIRLs were associated with HCC prognosis. **(B–C)** Lasso regression confirmed seven IIRLs. **(D–G)** LINC01871, AC011407.1, and AC006369.1 were independent protective factors for HCC, while LINC01094 was an independent risk factor for HCC.

Dividing the dataset into training and testing cohorts in a random fashion, we compared the baseline data of three cohorts (Table 1). The univariate Cox regression performed on training cohort revealed that eight IIRLs were relevant with HCC prognosis (Figure 3A). These genes were then subjected to LASSO regression, and seven IIRLs were discovered following LASSO regression (Figures 3B,C). We found out four IIRLs (LINC01871, AC011407.1, LINC01094, AC006369.1) by using multivariate Cox regression. LINC01871, AC011407.1, and AC006369.1 were protective factors for HCC, while LINC01094 was a risk factor for HCC (Figures 3D–G). The following risk score formula was used to produce an IIRLs prognostic signature (Table 2).

**TABLE 1:** The baseline data of three cohorts.

	Total (*n* = 369)	Test (*n* = 184)	Train (*n* = 185)	p-value
Age				
≤65	232 (62.87%)	124 (67.39%)	108 (58.38%)	0.0791
>65	136 (36.86%)	59 (32.07%)	77 (41.62%)	
Unknow	1 (0.27%)	1 (0.54%)	0 (0%)	
Gender				
Female	120 (32.52%)	68 (36.96%)	52 (28.11%)	0.0886
Male	249 (67.48%)	116 (63.04%)	133 (71.89%)	
Grade				
G1	55 (14.91%)	28 (15.22%)	27 (14.59%)	0.3318
G2	177 (47.97%)	86 (46.74%)	91 (49.19%)	
G3	120 (32.52%)	63 (34.24%)	57 (30.81%)	
G4	12 (3.25%)	3 (1.63%)	9 (4.86%)	
Unknow	5 (1.36%)	4 (2.17%)	1 (0.54%)	
Stage				
Stage I	171 (46.34%)	86 (46.74%)	85 (45.95%)	0.9712
Stage II	86 (23.31%)	44 (23.91%)	42 (22.7%)	
Stage III	83 (22.49%)	42 (22.83%)	41 (22.16%)	
Stage IV	5 (1.36%)	2 (1.09%)	3 (1.62%)	
Unknow	24 (6.5%)	10 (5.43%)	14 (7.57%)	
T				
T1	181 (49.05%)	93 (50.54%)	88 (47.57%)	0.8125
T2	94 (25.47%)	45 (24.46%)	49 (26.49%)	
T3	77 (20.87%)	35 (19.02%)	42 (22.7%)	
T4	13 (3.52%)	7 (3.8%)	6 (3.24%)	
Unknow	4 (1.08%)	4 (2.17%)	0 (0%)	
M				
M0	265 (71.82%)	132 (71.74%)	133 (71.89%)	0.6303
M1	4 (1.08%)	1 (0.54%)	3 (1.62%)	
Unknow	100 (27.1%)	51 (27.72%)	49 (26.49%)	
N				
N0	250 (67.75%)	121 (65.76%)	129 (69.73%)	0.1226
N1	4 (1.08%)	4 (2.17%)	0 (0%)	
Unknow	115 (31.17%)	59 (32.07%)	56 (30.27%)	

**TABLE 2:** Multivariate Cox results for IIRLs.

Id	Coef	HR	HR.95 L	HR.95 H	*p*-value	Risk
LINC01871	−0.3043	0.7377	0.543	1.0021	0.0516	Low
AC011407.1	−2.199	0.1109	0.0088	1.3988	0.089	Low
LINC01094	1.2429	3.4658	1.551	7.7444	0.0024	High
AC006369.1	−1.8701	0.1541	0.029	0.8186	0.0282	Low

Risk score = (1.2429*Exp LINC01094) -(0.3043*Exp LINC01871) -(2.199*Exp AC011407.1) -(1.8701*Exp AC006369.1).

### Assessment of an immune infiltration-related lncRNAs prognostic signature

**FIGURE 4:**
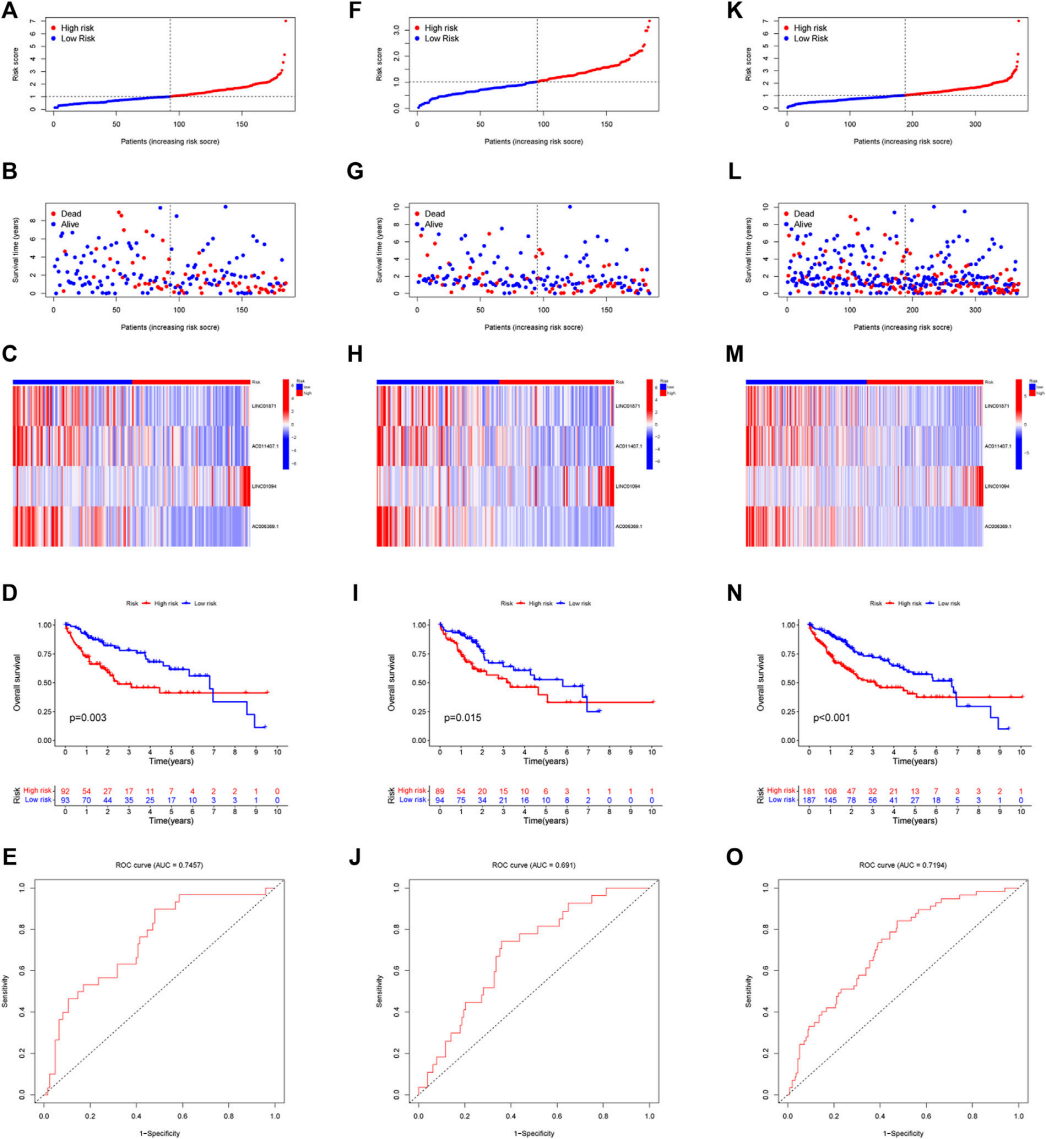
Modeling and verifying risk scores. **(A)** Distribution of risk scores among patients in training cohort. **(B)** A scatterplot of the relationship between OS and risk score of each person in training cohort. **(C)** Expression of four IIRLs in training cohort using heatmap. **(D)** The KM survival curve of training cohort. **(E)** The training cohort’s 1-year ROC curve’s area under the curve. **(F)** Distribution of risk scores among patients in testing cohort. **(G)** A scatterplot of the relationship between OS and risk score of each person in testing cohort. **(H)** Expression of four IIRLs in testing cohort using heatmap. **(I)** The KM survival curve of testing cohort. **(J)** The testing cohort’s 1-year ROC curve’s area under the curve. **(K)** Distribution of risk scores among patients in entire cohort. **(L)** A scatterplot of the relationship between OS and risk score of each person in entire cohort. **(M)** Expression of four IIRLs in entire cohort using heatmap. **(N)** The KM survival curve of entire cohort. **(O)** The entire cohort’s 1-year ROC curve’s area under the curve.

Each sample in the training cohort had its risk score determined by the risk score methodology. According to the median risk score (1.025), we classfied all patients into low-risk and high-risk (Figure 4A). A progressive decline in survival time and an increase in mortality were seen in conjunction with an increase in risk score (Figure 4B). Heatmaps were used to display the expression levels of four IIRLs (LINC01871, AC011407.1, LINC01094, and AC006369.1) in the training cohort (Figure 4C). The finding shows two groups had significantly different OS, according to the KM survival curve (*p* = 0.003) (Figure 4D). By time-dependent ROC curve, we predicted lncRNA biomarkers with an AUC of 0.7457 (Figure 4E). The signature was then tested on testing cohort and entire cohort to ensure its accuracy (Figures 4F–O). Throughout testing cohort and entire cohort, we found the high-risk group had a substantially shorter OS compared to the low-risk group. Moreover, the AUCs for testing cohort and entire cohort were 0.691 and 0.7194, which indicated that this prognostic signature has good stability.

### Clinical value of the prognostic signature

**FIGURE 5:**
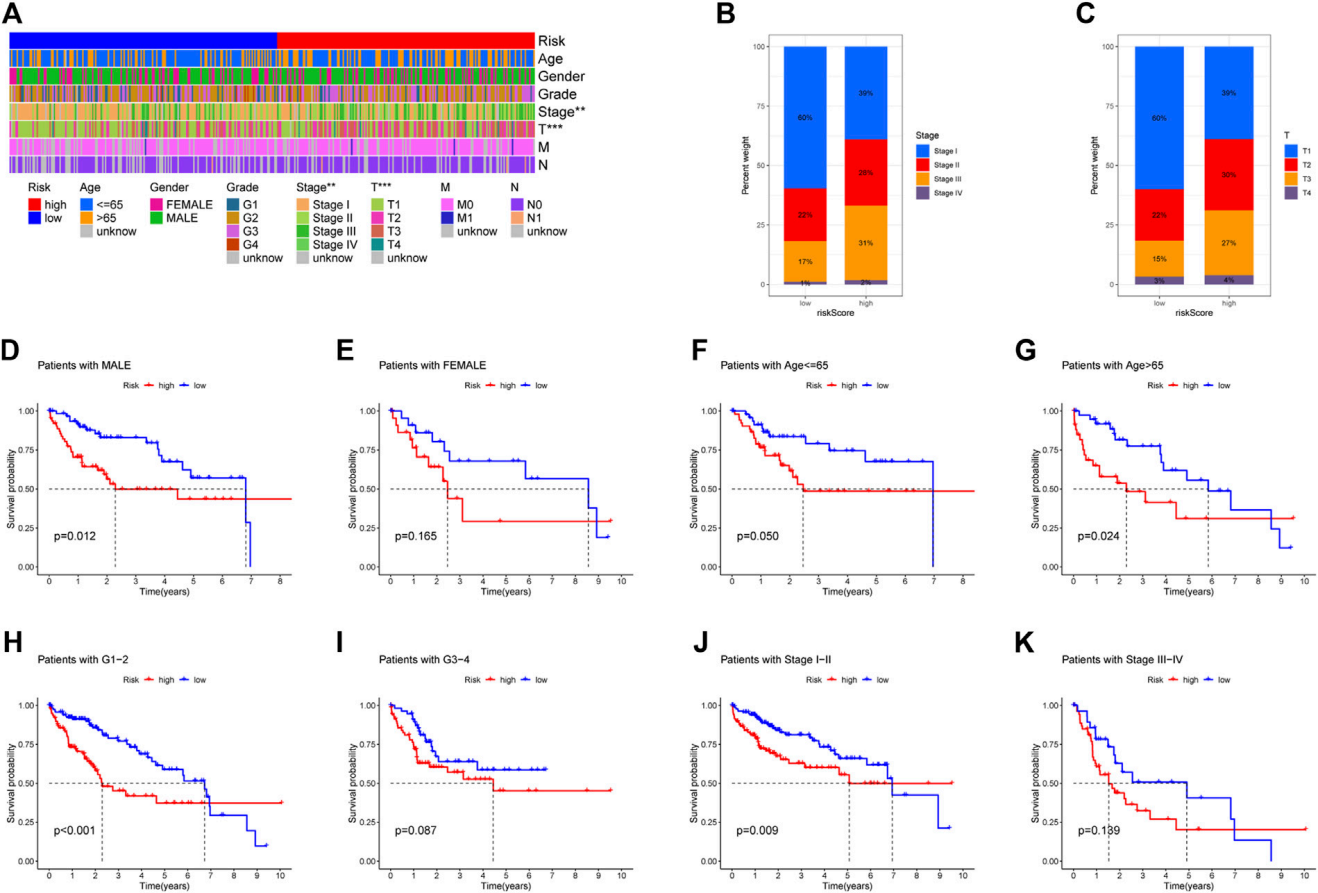
Comparison of overall survival (OS) between subgroups of individuals with different clinicopathological features. **(A)** The risk-clinical characteristic heatmap was used to visualize this relationship (∗*p* < 0:05, ∗∗*p* < 0:01, and ∗∗∗*p* < 0:001). **(B)** Distribution of AJCC stage in two groups. **(C)** Distribution of T-stage in two groups. **(D–E)** Subgroup of gender (male and female). **(F–G)** Subgroup of age (≤65 years and >65 years). **(H–I)** Subgroup of grade (G1, G2, G3, and G4). **(J–K)** Subgroup of AJCC stage (I, II III, and IV).

**FIGURE 6:**
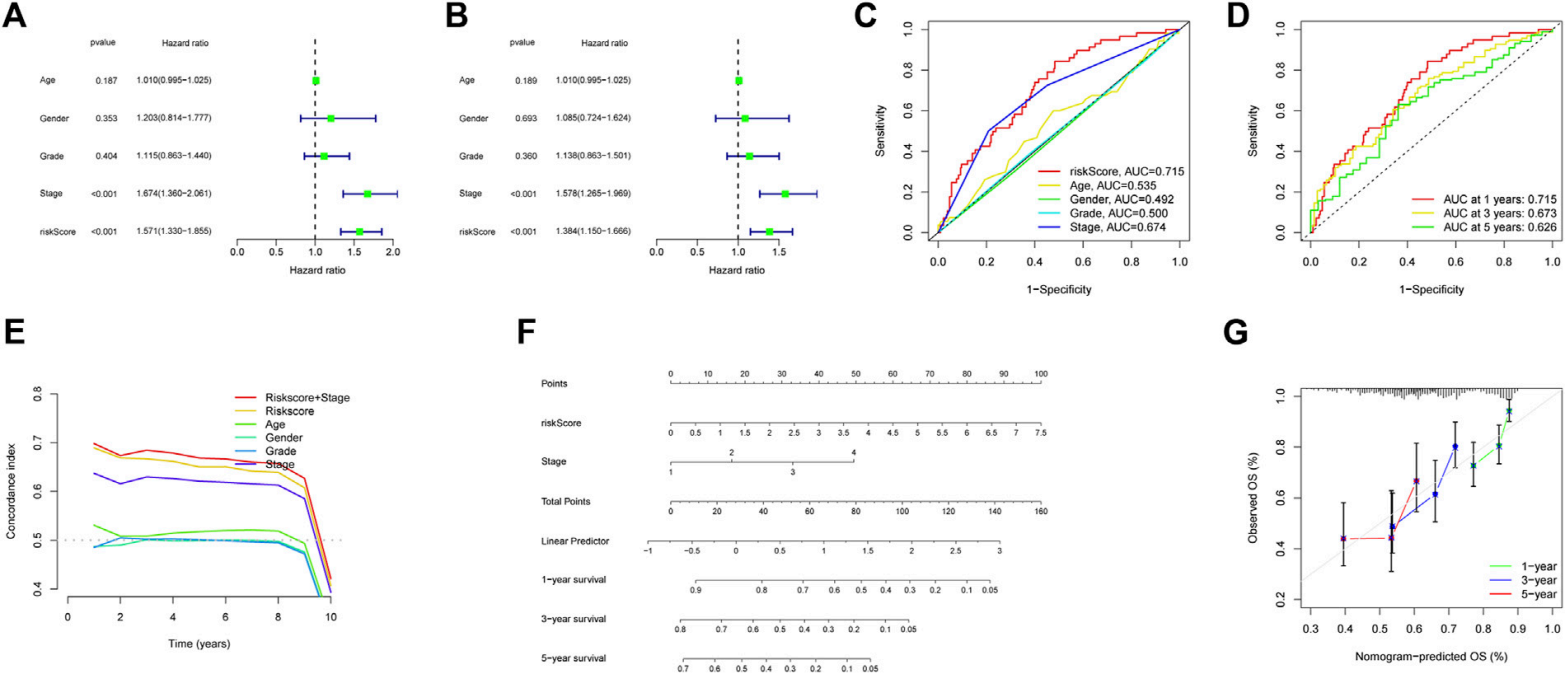
Building a Nomogram and Evaluating Its Performance. **(A)** The AJCC stage and risk score were shown to be associated with overall survival in a univariate Cox regression analysis (*p* < 0.05). **(B)** Multivariate Cox regression analysis confirmed the significance of AJCC stage and risk score as independent predictive markers of OS in HCC patients (*p* < 0.05). **(C)** According to the ROC curve, risk score was the most predictive metric. **(D)** The ROC curves of 1-year, 3-years, and 5-years risk score. **(E)** C-index of clinical factors. **(F)** Using risk score and AJCC stage, this nomogram estimate a patient’s survival rate at one, three, and 5 years after diagnosis with HCC. **(G)** Nomogram accuracy was shown by a calibration curve at one, three, and 5 years.

Figure 5A showed the heatmap of clinical information of patients in two groups and we compared the two groups to see whether there were differences in clinical information. The distributions of AJCC stage and T-stage were different for the high-risk and low-risk groups (Figures 5B,C). Except for three subgroups (subgroup of female, subgroup of G3 and G4, subgroup of stage III and stage IV), OS was shorter for patients in the high-risk group (Figures 5D–K). For HCC patients, we can see that the risk score was a significant risk factor connected to their prognosis by univariate Cox regression analysis (95% confidence interval (CI): 1.330–1.855, *p* < 0.001). In addition, AJCC stage (95%CI:1.360–2.061, *p* < 0.001) was also closely related to prognosis (Figure 6A). Risk score remained a significant factor in multivariate Cox regression (HR = 1.384, 95% CI: 1.150–1.666, *p* < 0.001, Figure 6B). By ROC analysis, we confirmed that the risk score had higher accuracy in predicting patients compared with other clinicopathological features (Figure 6C). Comparative ROC curves for risk scores at one, three, and 5 years were created (Figure 6D). The 1-year risk score had the best predictive accuracy.

### Building a nomogram and evaluating its performance

Based on risk score and AJCC stage, we made a nomogram by using multivariate Cox regression. When the index values for each AJCC stage and risk factor were added together, a prediction of survival at 1, 3, and 5 years could be made (Figure 6F). The concordance index shows strong stability in the nomogram (Figure 6E). The calibration curve also demonstrated that the anticipated survival lengths at 1, 3, and 5 years were consistent with the reference line (Figure 6G).

### Functional enrichment analysis

**FIGURE 7:**
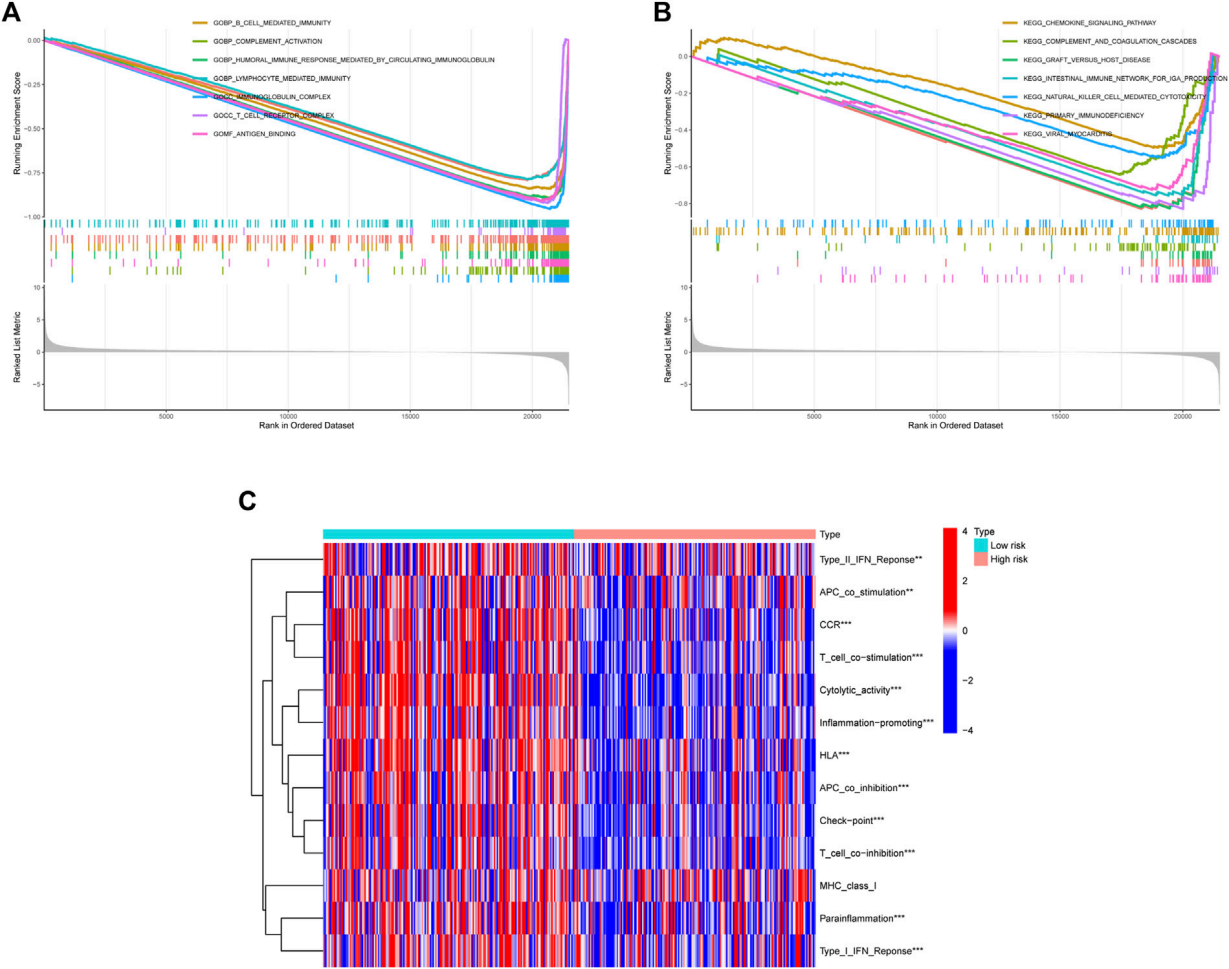
The results of enrichment analysis. **(A)** Enrichment analysis of GO. **(B)** Enrichment analysis of KEGG. **(C)** ssGSEA immune function enrichment analysis.

We used the GSEA function to perform GO and KEGG enrichment analysis. GO analysis showed that they were significantly enriched in B cell mediated immunity, complement activation, humoral immune response mediated by circulating immunoglobulin, lymphocyte mediated immunity, immunoglobulin complex, T-cell receptor complex, and antigen binding (Figure 7A). According to KEGG analysis, they were significantly enriched in the following pathways: chemokine signaling pathway; complement and coagulation cascades; graft *versus* host disease; intestinal immune network for IgA production; natural killer cell mediated cytotoxicity; primary immunodeficiency; viral myocarditis (Figure 7B).

### Immune correlation analysis

**FIGURE 8:**
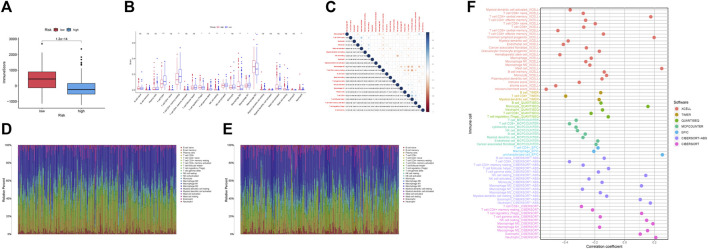
Immunological correlation and immune differential studies were performed on two groups of HCC patients. **(A)** Boxplots showed the ImmuneScore is significantly higher in low-risk group. **(B)** The differential analyses of immune cells for two groups. **(C)** The correlation plot of immune cells. **(D)** Distribution of 22 infiltrating immune cell subtypes in the low-risk group. **(E)** Distribution of 22 infiltrating immune cell subtypes in high-risk group. **(F)** Relationship between different immune cells and risk score.

The ssGSEA immune function scores were performed based on each sample. With the exception of MHC class I, low-risk group had better immune function ratings than high-risk group (*p* < 0.05) (Figure 7C). The number of individuals in low-risk group had a high ImmuneScore than in high-risk group. (Figure 8A). Using the CIBERSORT algorithm, we were able to determine the percentage of 22 different types of immune cells present in HCC patients. The boxplot clearly displayed the dissimilar distributions of immune cells between two groups. There were less CD8^+^ T-cell, resting memory CD4^+^ T-cell, Gamma delta T-cell, activated NK cells, and classically activated macrophages in high-risk group compared to low-risk group (Figure 8B). Figure 8C showed the correlation between various immune cells. Figures 8D,E demonstrated the distribution of 22 immune cells in two groups. When we examined the correlation between immune cells and risk score using various algorithms, the findings revealed that the majority of immune cells had a poor relationship with risk score (Figure 8F).

### Drug prediction

**FIGURE 9:**
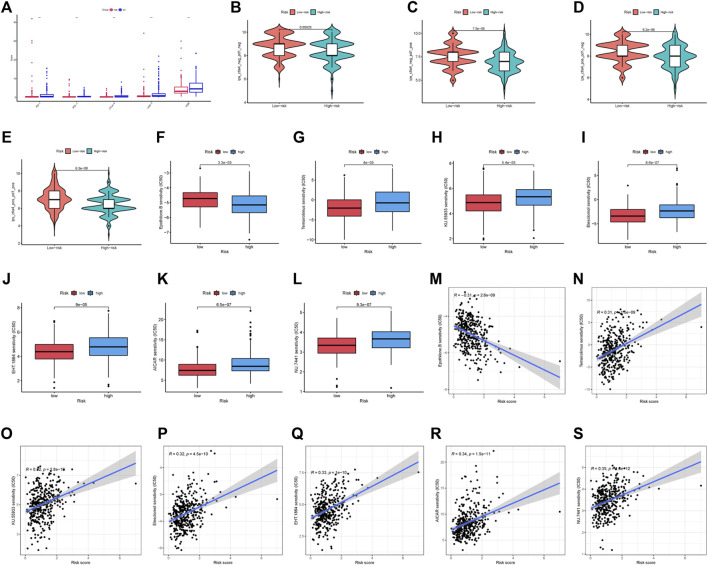
Drug prediction and risk stratification correlated with the efficacy of immunotherapy. **(A)** Five immune checkpoints and their expression in two groups (∗*p* < 0:05, ∗∗*p* < 0:01, and ∗∗∗*p* < 0:001). **(B)** IPS of high-risk and low-risk groups. **(C)** IPS- PD1 of high-risk and low-risk groups. **(D)** IPS- CTLA4 of high-risk and low-risk groups. **(E)** IPS- PD1 + CTLA4 of high-risk and low-risk groups. **(F–L)** IC50 of seven drugs (epothilone.B, temsirolimus, KU.55933, elesclomol, EHT. 1864, AICAR, NU.7441) in two groups. **(M–S)** Relationship between risk score and IC50 of seven drugs (epothilone.B, temsirolimus, KU.55933, elesclomol, EHT. 1864, AICAR, NU.7441).

The low-risk group had significantly greater expression levels of immune checkpoint genes (Figure 9A). The association of IPS with high-risk and low-risk groups was investigated. We used IPS, IPS- PD1, IPS-CTLA4, and IPS- PD1 + CTLA4 to evaluate the potential of risk scores application. The IPS, IPS-PD1, IPS-CTLA4, and IPS- PD1 + CTLA4 were different in two groups (*p* < 0.05) (Figures 9B–E). Based on the CGP database, drug IC50 information was obtained to predict treatment response, and epothilone. B may be a potential therapeutic drug for patients in high-risk groups (Figures 9F, M). Six drugs (temsirolimus, KU.55933, elesclomol, EHT. 1864, AICAR, NU.7441) were screened as potential therapeutic drugs for patients in low-risk group (Figures 9G–L, N–S).

## Discussion

With the deepening of research on hepatocellular carcinoma and the improvement of therapeutic methods, the 5-years survival rate of patients is still low. TME is linked to tumor development and response to treatment, and immunotherapy is evolving into a novel approach to treating high-grade cancers. At present, a variety of ICIs have been applied to the second-line treatment of HCC systemic therapy, but they are only effective in hepatocellular carcinoma patients with immune response. For that reason, how to predict outcomes for people with HCC and guide the signature of immunotherapy has become increasingly crucial. LncRNAs are key players in the tumor immune system and can be used as potential prognostic biomarkers. In our study, we applied a combination of univariate Cox, LASSO, and multivariate Cox regression to establish an immune infiltration-related lncRNAs prognostic signature. The prediction performance of this signature was high. Patients classified as low risk have a longer expected survival time than those of high risk. Our final tally of four IIRLs suggests they may serve as both prognostic indicators and therapeutic targets for hepatocellular carcinoma.

Among the four IIRLs risk model, the biological mechanisms of LINC01871, AC011407.1, and AC006369.1 have not been reported to date. Based on bioinformatics analysis, some prognostic models constructed suggested that LINC01871 was related to immune response (Chen et al., 2020; Ma et al., 2020; Mathias et al., 2021; Wang et al., 2021). To exert their functions, lncRNAs are used as signals, decoys, scaffolds, guides, or enhencers (Wang and Chang, 2011; El Khodiry et al., 2018). Therefore, the roles of these three lncRNAs in hepatocellular carcinoma should be further studied in the near future. Several studies have indicated that LINC01094 can act as an effective miR-577 sponge to promote the proliferation, invasion and metastasis of various tumors (Xu et al., 2020a; Dong et al., 2020; Luo et al., 2021). Targeting the miR-577/CHEK2/FOXM1 axis, LINC01094 promotes radioresistance in clear cell renal cell carcinoma (ccRCC) (Jiang et al., 2020). Apart from acting as an effective miR-577 sponge, LINC01094 can competitively bind to a variety of microRNAs to promote tumor progression (Xu et al., 2020b; Li and Yu, 2020; Zhu et al., 2020; Wu et al., 2021b; Chen et al., 2021; Liu et al., 2022). From our study, the expression of LINC01094 is clearly elevated in high-risk group compared to low-risk group, which may promote the progress of HCC. Although LINC01094 is related to the progression of ccRCC, glioblastoma, and ovarian cancer (Xu et al., 2020b; Li and Yu, 2020; Chen et al., 2021), still no studies have shown a relationship between LINC01094 and the progression of HCC. Additional trials are needed to confirm whether or not LINC01094 may accelerate HCC development. The biological roles of lncRNAs in HCC including cell proliferation, cell death, metabolic reprogramming, angiogenesis, metastasis, inflammation and tumour immunity (Yu et al., 2018; Xie et al., 2021). The roles of lncRNAs in tumor immunity can be classified into the following five points (Yu et al., 2018): (Bray et al., 2018) bidirectional regulation of antigen release (Chidambaranathan-Reghupaty et al., 2021); participating in antigen presentation (Kulik and El-Serag, 2019); regulating immune cell differentiation during immune priming and activation (Llovet et al., 2021a); influencing immune cells migration and T-cell infiltration (Jemal et al., 2017); affecting the recognition and killing of cancer cells. At present, there are no relevant studies on tumor immunity of these four IIRLs in HCC. For this reason, we think that these IIRLs can be deeply studied from five aspects of tumor immunity above.

GSEA enrichment results were mainly in immune related pathways. Compared to low-risk group, the expression of these pathways was considerably lower in high-risk group, according to the negative enrichment score. The results of immune function enrichment also demonstrated that the immune function of high-risk group decreased accordingly. Myeloid-derived suppressor cells (MDSCs), tumor-associated macrophages (TAMs), tumor associated neutrophils (TANs), regulatory T-cell (Treg) can achieve immune escape through immune suppression, thus promoting tumor development (Fu et al., 2019; Lawal et al., 2021). MDSCs can achieve immunosuppression by producing immunosuppressive factors (such as arginase, inducible nitric oxide synthase, IL-10, TGF-β) to inhibit cytotoxic T-cell and NK cell function (Gabrilovich and Nagaraj, 2009; Masucci et al., 2019; Lawal et al., 2021). There are two distinct phenotypes among TAMs, M1, and M2. The M2 phenotype promotes tumor initiation, progression, and metastasis *via* many pathways (Qian and Pollard, 2010; Yao et al., 2018). By activating the TLR4/TRIF/NF-B signaling pathway, HCC cells encourage the immunological evasion of HCC by boosting IL-1 secretion of TAMs (Zhang et al., 2018). According to their polarizing effects, Tans can be classified as either anti-tumor (N1) or pro-tumor (N2) phenotypes, and the degree of invasion of Tans is strongly correlated with tumor growth (Nicolas-Avila et al., 2017; Fu et al., 2019). Inducing apoptosis in CD8^+^ T lymphocytes through nitric oxide generation mediated by tumor necrosis factor alpha (TNF-α) is how TANs accomplish their immunosuppressive effects (Michaeli et al., 2017). By increasing AP-1/NF-AT1 axis activity, Tregs aid HCC in suppressing the immune system (Jiang et al., 2017). Through downregulating the synthesis and secretion of components like granzyme, perforin, TNF-α and IFN-γ, Tregs influence CD8^+^ T-cell production and cytotoxicity (Fu et al., 2007; Hoechst et al., 2008; Huang et al., 2012). Compared with the low-risk group, our study showed that the expression of Tregs, macrophages and neutrophils in high-risk group was increased. This indicated that the immune function of the high-risk group may be suppressed, which was consistent with the results of immune function analysis. And there were relatively few CD8^+^ T-cell in the high-risk group. According to a research, immunotherapy is ineffective against tumors lacking CD8^+^ T-cell infiltration and they have a poorer prognosis (Gajewski, 2015). Those in low-risk group showed greater levels of immune infiltration, higher expression of immune checkpoint genes, and higher IPS scores when compared to patients in high-risk group. Immunotherapy was more likely to be beneficial for patients in low-risk group.

We combined patient prognosis analysis with IIRLs to design a signature which can predict survival rate and therapeutic drugs for the high-risk and the low-risk groups. Though having made some progress, we acknowledged that our research has certain limitations. First, the main data set we collected from TCGA database, lacks an external validation set; Other data sets should be acquired and analyzed to further validate the signature. Second, we did not elucidate the mechanism and function of IIRLs, which deserve further study in the future. Third, no clinical data were found to support the clinical feasibility of treatment drugs in two groups.

## Conclusion

We created a signature of immune infiltrationrelated lncRNAs that may be utilized as a novel biomarker to predict HCC development. This signature contributes to a deeper understanding of the correlation between immune infiltration and tumor progression. It is expected to be further utilized in future clinical practice.

## Data Availability

The original contributions presented in the study are included in the article/Supplementary Material, further inquiries can be directed to the corresponding author.
